# Supplementary figures and images for: Functionally impaired isoforms regulate TMPRSS6 proteolytic activity
Source: PLoS One. 2022 Aug 31;17(8):e0273825. doi: 10.1371/journal.pone.0273825 (PMC9432768; doi:10.1371/journal.pone.0273825)

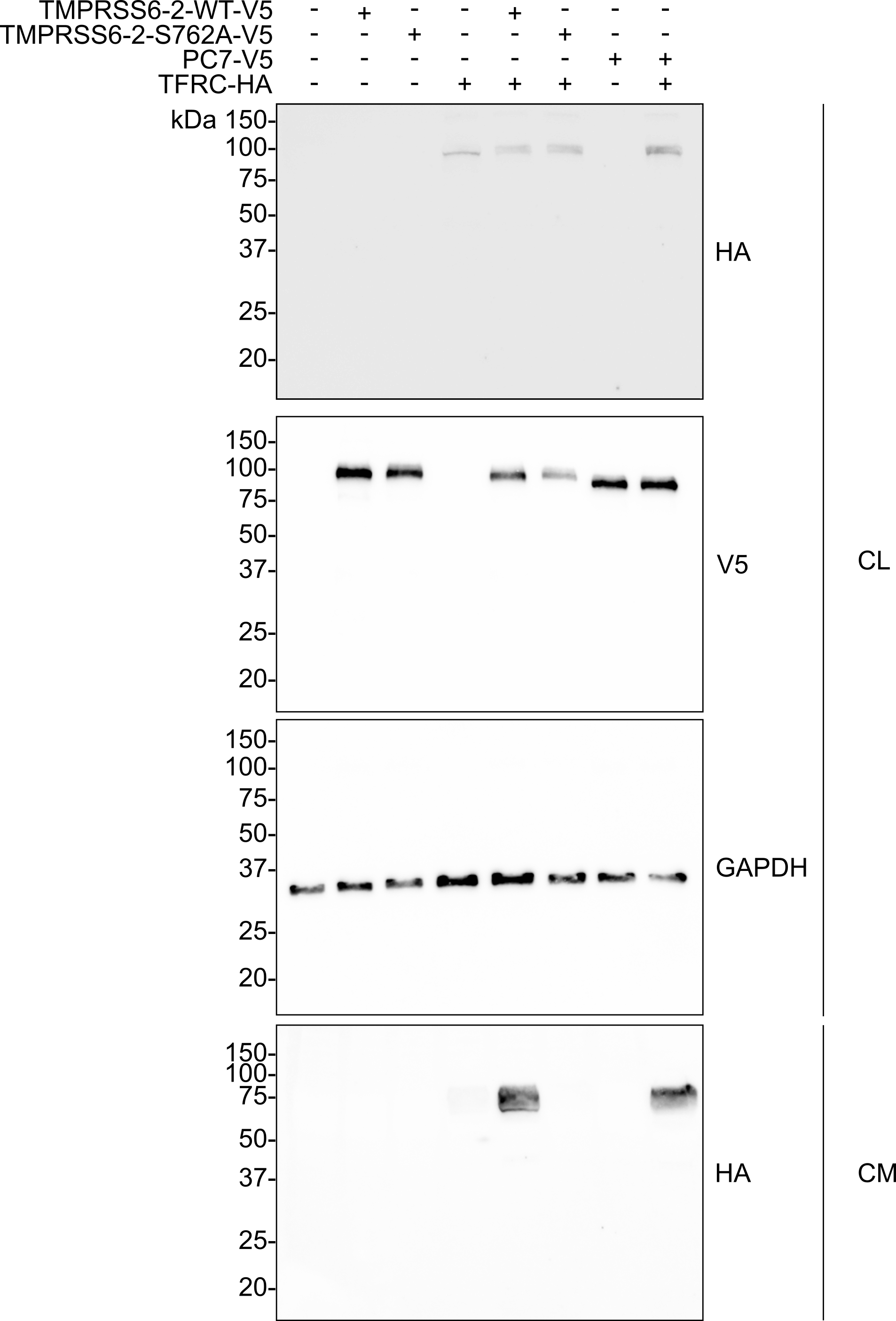

Supplement: S1 Fig — TfR1-HA was transfected either with V5-tagged TMPRSS6 isoform 2 WT (active, TMPRSS6-2-WT-V5), catalytically inactivated TMPRSS6 isoform 2 (TMPRSS6-2-S762A-V5) or proprotein convertase 7 (PC7-V5). Expression was detected in the cell lysate (CL) and cell media (CM) (n = 3). (TIF) [file pone.0273825.s001.tif]

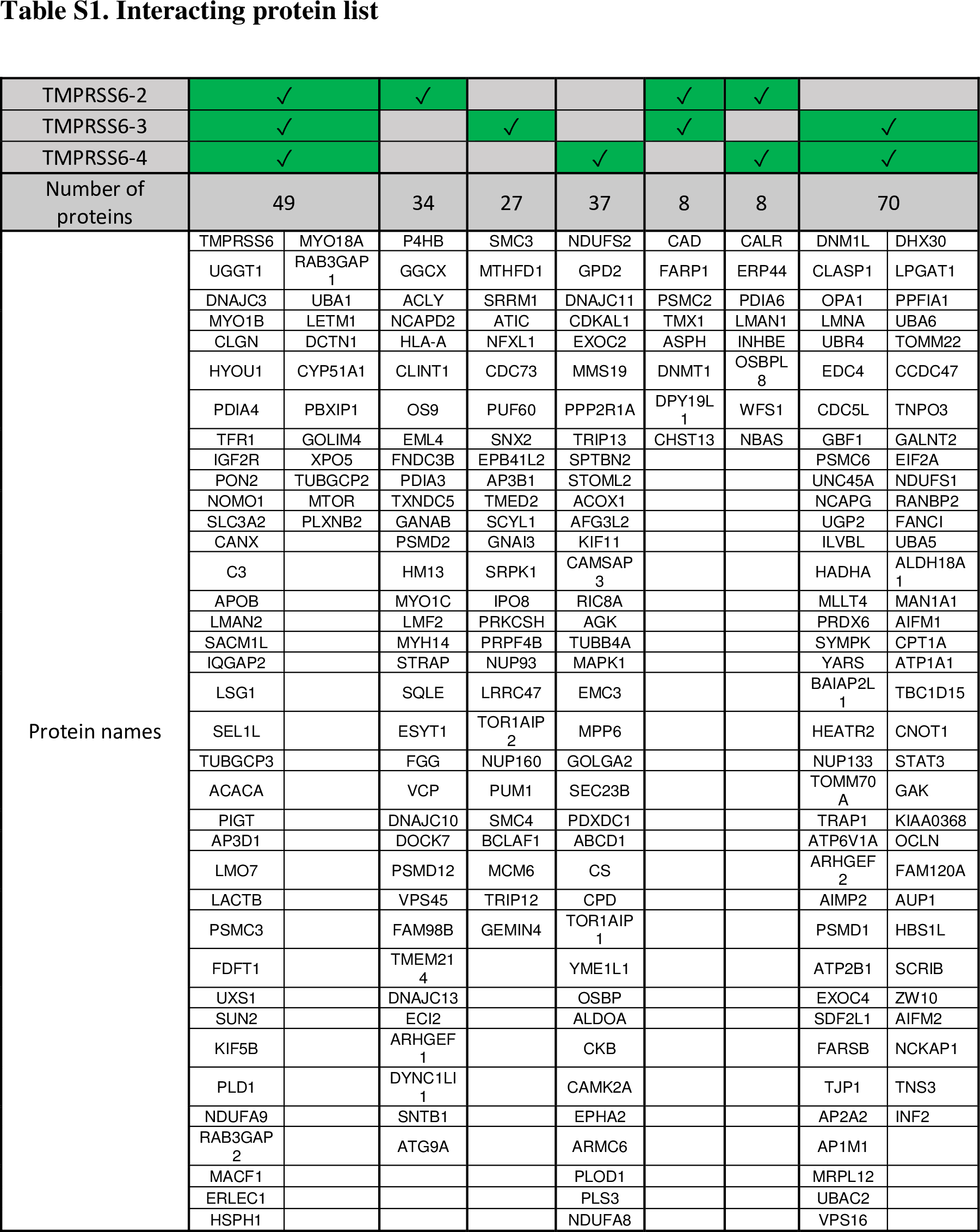

Supplement: S1 Table — (TIF) [file pone.0273825.s002.tif]
